# Genome-Wide Analysis of Gene and Protein Expression of Dysplastic Naevus Cells

**DOI:** 10.1155/2012/981308

**Published:** 2012-11-28

**Authors:** Linda Gao, Frans A. van Nieuwpoort, Jacoba J. Out-Luiting, Paul J. Hensbergen, Femke A. de Snoo, Wilma Bergman, Remco van Doorn, Nelleke A. Gruis

**Affiliations:** ^1^Department of Dermatology, Leiden University Medical Center, Einthovenweg 20, 2333 ZC Leiden, The Netherlands; ^2^Department of Parasitology, Leiden University Medical Center, Albinusdreef 2, 2300 RC Leiden, The Netherlands; ^3^Center for Human and Clinical Genetics, Leiden University Medical Center, Albinusdreef 2, 2300 RC Leiden, The Netherlands

## Abstract

Cutaneous melanoma, a type of skin tumor originating from melanocytes, often develops from premalignant naevoid lesions via a gradual transformation process driven by an accumulation of (epi)genetic lesions. These dysplastic naevi display altered morphology and often proliferation of melanocytes. Additionally, melanocytes in dysplastic naevi show structural mitochondrial and melanosomal alterations and have elevated reactive oxygen species (ROS) levels. For this study we performed genome-wide expression and proteomic analysis of melanocytes from dysplastic naevus (DNMC) and adjacent normal skin (MC) from 18 patients. Whole genome expression profiles of the DNMC and MC of each individual patient subjected to GO-based comparative statistical analysis yielded significantly differentially expressed GO classes including “organellar ribosome,” “mitochondrial ribosome,” “hydrogen ion transporter activity,” and “prefoldin complex.” Validation of 5 genes from these top GO classes revealed a heterogeneous differential expression pattern. Proteomic analysis demonstrated differentially expressed proteins in DNMC that are involved in cellular metabolism, detoxification, and cytoskeletal organization processes, such as GTP-binding Rho-like protein CDC42, glutathione-S-transferase omega-1 and prolyl 4-hydroxylase. Collectively these results point to deregulation of cellular processes, such as metabolism and protein synthesis, consistent with the observed elevated oxidative stress levels in DNMC potentially resulting in oxidative DNA damage in these cells.

## 1. Introduction

Cutaneous melanoma is a malignant tumour that originates from melanocytes, the pigment producing cells of the skin. Melanoma development is a multistep process driven by the acquisition of genetic and epigenetic abnormalities. Although a subset of melanomas develop in normal skin de novo, in many instances premalignant naevoid pigmented lesions can be discerned in melanoma formation. Approximately a third of melanomas develop from a precursor naevus that is most commonly dysplastic [1]. Consistently, individuals with dysplastic naevi are at increased risk of developing melanoma [2].

Dysplastic naevi can progress into radial and subsequently vertical growth phase melanomas [3, 4]. The complex multistage development process of melanoma is characterized by various morphological, cellular, and biochemical alterations. Histopathologically dysplastic naevi show characteristic morphological alterations, including proliferation and variable atypia of epidermal melanocytes, formation of irregular cell nests in the epidermis and basement membrane zone, and the interconnection of these nests and layers (bridging). Melanocytes in dysplastic naevi (DNMC) furthermore exhibit morphological alterations in melanosomes and mitochondria, similar to those observed in melanoma cells [5]. We have previously shown that DNMC in comparison with normal melanocytes show higher pheomelanin, iron, and calcium levels resulting in elevated reactive oxygen species (ROS) levels [6, 7]. The diminished levels of antioxidant enzymes in DNMC further highten ROS levels and reinforce chronic oxidative stress in these cells [8, 9].

In the present study we compared the gene expression and protein expression patterns of melanocytes from dysplastic naevus and from normal skin of 18 individuals. From a surgically removed dysplastic naevi and adjacent normal skin, melanocytes were isolated and briefly cultured for gene expression analysis using whole genome expression arrays and proteome analysis by peptide mass fingerprinting. Gene ontology-based gene expression analysis and protein expression analysis revealed differentially expressed pathways involved in cellular metabolism, detoxification, and cell shape organization. These findings appear to signify deregulated processes that all together may contribute to an increase in the levels of reactive oxygen species and oxidative stress, as is often observed in DNMC. The resultant oxidative DNA damage is an endogenous mutagenic force that might contribute to melanoma development.

## 2. Materials and Methods

### 2.1. Cell Culture

After approval by the Medical Review Board of Leiden University Medical Center and patient consent, 18 clinically atypical naevi were excised from 18 different patients. All 18 samples were confirmed after pathologist review as dysplastic naevus. The elliptical incision consisted of the dysplastic naevus in the central part and normal skin at the tips. To ensure separate isolation of melanocytes from the dysplastic naevus and from adjacent normal skin, the central part and tip of each surgical specimen were first divided before further processing. For isolation of the melanocytes from the dysplastic naevus and normal skin from the epidermal compartment, we used an established protocol described previously [6, 7]. Briefly, after removal of subcutaneous fat, epidermis and dermis were separated by overnight incubation with dispase grade II (Boehringer Mannheim Inc, Indianapolis, IN). The epidermis was trypsinized to obtain a single cell suspension using 0.25% trypsin. The cells from this single cell suspension were plated in Ham's F10 melanocyte medium supplemented with penicillin (100 U/mL), streptomycin (100 U/mL), L-glutamine (Invitrogen, Breda, The Netherlands), 1% Ultroser G (BioSepra, Fremont, CA), Endothelin-1 (5 ng/mL), basic-FGF (5 ng/mL), cholera toxin (30 *μ*g/mL), IBMX (33 *μ*M), and TPA (8 nM, Sigma-Aldrich, Zwijndrecht, The Netherlands). This melanocyte medium contains ingredients that are toxic to keratinocytes present in the epidermal cell suspension. Under these conditions pure melanocyte cultures are obtained at 10 days after start of culture. Purity of these melanocyte cultures was confirmed by microscopic examination of cell morphology by clockwise assessment of the culture plate surface; after 10 days all cells invariably had the dendritic, star-shaped or spindle-like morphology of melanocytes and no cells with epithelioid morphology were present in the culture dish. Additional stainings of representative melanocyte cell cultures with melanocytic cell markers HMB45 and Melan-A confirmed the purity of these melanocyte cultures. Melanocytes isolated from dysplastic naevus (DNMC) and from normal skin (MC) were cultured for a maximum of 6 passages to ensure logarithmic growth and keep the time in culture as short as possible. To minimize the variation introduced by cell culture on gene and protein expression, all MC and DNMC patient samples underwent identical treatment in culture and were harvested at the same passage number with the same harvesting methods.

### 2.2. Sample Preparation for Microarrays and Peptide Mass Fingerprinting

RNA was isolated from melanocytes with the RNeasy Mini Kit (Qiagen, Venlo, Netherlands). Antisense amplified RNA (aRNA) was generated with the MessageAMP II aRNA Kit (Ambion, Austin, TX) for hybridization to the Human Genome U133 Plus 2.0 Array (Affymetrix, Santa Clara, CA) following manufacturer's protocol. RNA concentrations were determined by nanodrop measurements and RNA quality control was performed prior to hybridization by Lab-on-a-chip on Agilent's Bioanalyzer. RNA quality was further confirmed by posthybridization quality control measurements performed as part of the Affymetrix microarray analysis, including normalization scaling factor across the microarrays and cRNA transcript integrity as assessed by 3′ to 5′ probe set signal intensity ratios, as routinely performed at the Leiden Genome Technology Center. Protein was isolated from melanocyte cell pellets using TRIzol reagent (Invitrogen); protein concentrations were determined by the Pierce BCA Protein Assay Kit. Protein samples (10 *μ*g) were labelled with 1000 pmol fluorescence amine-reactive cyanine dyes for two-dimensional difference gel electrophoresis (2D DIGE) following manufacturer's protocol (Amersham-Pharmacia Biotech).

### 2.3. Statistical Analysis of Gene Expression Data

Probe set signal intensities derived from microarray scans were subjected to the robust multiarray average (RMA) algorithm as previously described [10]. This algorithm results in background-corrected, quantile normalized and log(base2)-transformed final probe intensities that serve as measures of gene expression; this log scale measure of expression will be further referred to as “(gene) expression value.” To identify genes that were differentially expressed in DNMC when compared to MC, a statistical analysis method (random variance model (RVM)-based *t*-test) suited for detection of gene expression differences in microarray experiments comprising relatively smaller sample sizes was used [11], while controlling the number and proportion of false discoveries with a multivariate permutation test as described previously [12]. Additional hierarchical clustering based on Euclidean distance was performed; clustering reproducibility was assessed using calculated *R* (reproducibility) and *D* (discrepancy) indices [13].

### 2.4. Functional Class Scoring Based on Gene Ontology Annotations

All gene ontology annotations were obtained through http://www.geneontology.org/. Gene ontology (GO) classes with fewer than 5 and more than 100 genes represented in the array data were not considered in order to exclude very small and very large classes. The DNMC sample of one individual patient was compared to the paired MC sample, in order to assess whether differential expression of GO classes existed across all microarrays between the paired melanocyte sample set. Functional class scoring, based on semisupervised gene expression data analysis, was performed according to the statistical method as previously described [14]. This method does not require prior gene selection and *P* values are assigned based on interrogated genes in a GO class.

### 2.5. 2D DIGE and Mass Spectrometry Analysis

2D DIGE was performed as previously described [15]. Dye swap experiments were performed to check for labelling differences between Cy3 and Cy5. Gels were scanned in low-fluorescent glass plates with a Typhoon 9400 scanner (GE Health care UK Ltd., Buckinghamshire, UK). TIFF images of the gels were analysed with Delta2D v3.4 software (Dodecon, Greifswald, Germany). Spots were quantified and normalised based on total spot volume of one gel. The matched spot volumes were subsequently normalised across the whole set of gels and normalised spot volumes were compared between images in the set. Statistical significant differences between normalised spot volumes were determined using the Welch-modified Student's *t*-test and step-down Westfall and Young method as correction for the false discovery rate. Protein spots of interest were excised from the gel for analysis on a MALDI-TOF/TOF instrument (Ultraflex, Bruker, Germany). All mass fingerprint and MS/MS data were searched against the human protein database with the Mascot program (Matrix Science, Boston, MA).

### 2.6. Quantitative Real-Time PCR

cDNA was generated from DNase-treated RNA (1 *μ*g) with the iScript cDNA synthesis kit (BioRad, Hercules, CA). qRT-PCR was performed with iQ SYBR Green Supermix on a MyiQ Real-Time PCR Detection System (BioRad). *RPS11* served as reference gene in experiments. qRT-PCR expression data is presented as mean of two measurements. 

## 3. Results

### 3.1. Microarray Analysis of Melanocytes from Dysplastic Naevus and Normal Skin

**Figure 1:**
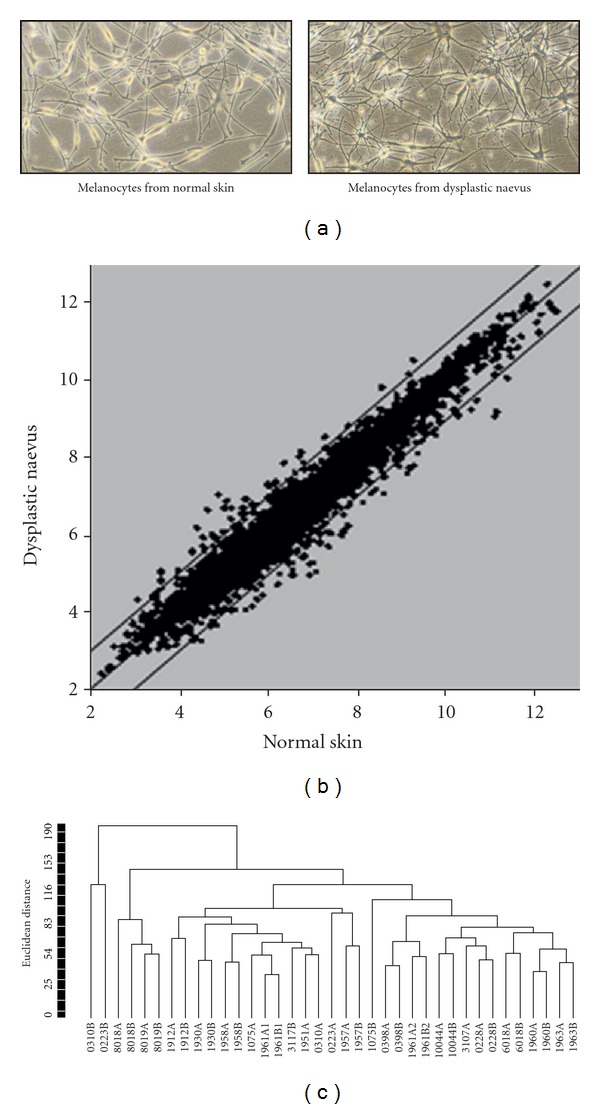
(a) Photos (4x magnification) taken from cultures of melanocytes derived from normal skin (left, MC) and dysplastic naevus (right, DNMC), representative of the MC and DNMC cultures of all 18 patients from which each of the melanocyte cultures were derived. (b) Scatter plot with average-log expression ratio's within one DNMC sample plotted against the average-log ratio's within the paired MC sample from one representative patient sample set. Each dot represents an interrogated probeset. The diagonal lines in the graph are outlier lines that indicate a 2-fold difference in the geometric mean of the expression ratio's between the DNMC and MC sample. (c) Clustering dendrogram of the expression values of all melanocyte samples. The vertical axis indicates the Euclidean distance with complete linkage as a measure for the extent of similarity between the samples. The horizontal axis contains all samples; A-labeled sample numbers, melanocytes derived from normal skin; B-labeled sample numbers, melanocytes derived from dysplastic naevus. The calculated *R*-index (0.997) and *D*-index (0.02) showed that the clusters were highly reproducible.

Previously it was demonstrated that melanocytes isolated from dysplastic naevus (DNMC) show differences in morphology, pigmentation, and growth abilities when compared to melanocytes isolated from normal skin (MC) in culture [7]. For this study paired DNMC and MC were obtained from dysplastic naevi and normal skin of 18 patients. Similarly we observed that the cultures of dysplastic naevus cells differed in morphology from that of normal melanocytes; Figure 1(a) displays a representative picture from melanocyte cultures derived from either normal skin (left, MC) or from dysplastic naevus (right, DNMC), showing that the MC have more equally-shaped dendrites as compared to the DNMC that consistently show a distinct stretched and thinner dendrite morphology. Microarray gene expression profiling was performed on these 18 paired melanocytic cell sets to gain insight into differences between these melanocyte sample groups on molecular level. Analysis of Affymetrix U133 plus 2.0 microarray data revealed that melanocytes from dysplastic naevi and from normal skin expressed 45% of the interrogated genes at a measurable level.  Figure 1(b) depicts a scatter plot where the expression ratio's of a representative sample set of DNMC and MC of one patient is plotted, indicating that gene expression differences between the DNMC and MC of the same patient were generally modest. Except for two samples, hierarchical clustering of the microarray data showed that the DNMC samples resembled their autologous normal counterparts more closely than they resemble the DNMC of another individual (Figure 1(c)). Of note, samples 0310B and 0223B clustered separately from the remaining samples; this distinction was not reflected in a different morphology of these cell samples, since cell morphology across the 18 DNMC samples was homogeneous without notable differences between the samples. In addition, all dysplastic naevi included in this study were histopathologically very similar and also in this respect samples 0310 and 0223 did not significantly differ from other patient samples.

We considered the possibility that in our melanocyte sample sets, smaller but parallel effects among groups or sets of genes may exist that act together within pathways. In this way we would not so much identify individual marker genes differentially expressed by DNMC, but rather obtain an impression of dysregulated biological processes in DNMC. We aimed to uncover genes that were highly relevant when placed in a larger network of genes that interact with it, even though showing small differential expression individually. We proceeded to analyze for each pair of DNMC and MC from a single patient, which functional groups of genes were differentially expressed. The top GO classes identified by functional class scoring as differentially expressed in DNMCs when compared to MCs, are listed in Table 1(a). Among the top represented GO classes were “organellar ribosome,” “mitochondrial ribosome,” “hydrogen ion transporter activity,” “prefoldin complex,” and “cAMP-dependent protein kinase regulator activity.” Table 1(b) presents the details of the top 5 GO classes that are differentially expressed between 18 sets of DNMCs and MCs. The genes in each GO class all had a *P* value < 0.05. Additional comparative statistical analysis of the gene expression data between the entire group of 18 DNMCs and 18 normal MCs did not yield significantly differentially expressed genes (data not shown).

### 3.2. Validation of Genes from Differentially Expressed GO Classes

**Figure 2:**
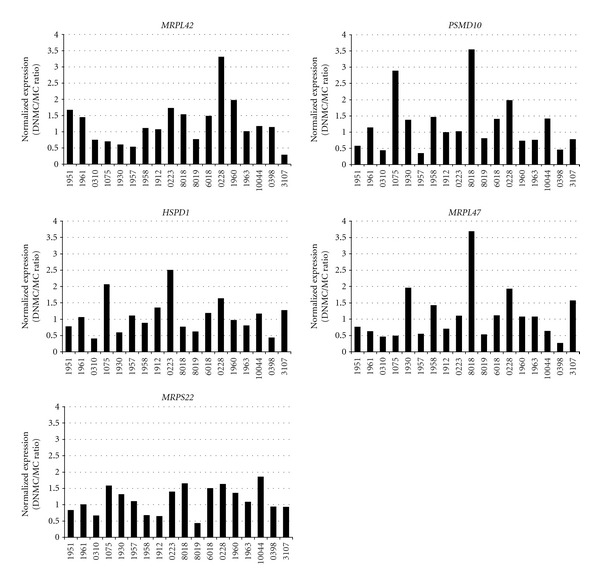
qPCR results across the 18 paired melanocyte sample groups for each of the five validated genes of the GO-based statistical analysis. Expression was calculated by taking the ratio between DNMC and the corresponding MC per patient sample set. *RPS11* served as reference gene in qPCR experiments performed in duplicate. Values <1: gene expression is lower in DNMCs when compared to normal skin-derived melanocytes.

Not all genes belonging to a certain GO class will necessarily exhibit differential expression between the DNMC and MC samples. To subsequently validate and assess whether individual genes from the identified GO classes are differentially expressed in DNMC as compared to MC, we selected 14 genes that were among the top 4 GO classes and analyzed expression levels by qPCR in the sample groups.  Table 2 provides an overview of the 14 selected genes for validation and the corresponding qPCR primer sequences.  Figure 2 shows the qPCR results for 5 of these validated genes (*MRPL42*, *PSMD10*, *HSPD1*, *MRPL47*, and *MRSP22*) with expression depicted as the ratio of gene expression in the DNMC to the corresponding expression in MC (vertical axis) plotted per sample for all 18 samples (horizontal axis). Differential gene expression seemed to vary per patient, as gene expression was either increased or decreased in DNMC as compared to MC for all interrogated paired melanocyte sample sets; this pattern was observed for all five genes. Some specific patient samples showed a higher increase in expression than other sample sets that also showed increased expression (sample 8018; expression *PSMD10 *and *MRPL47*), but a consistent differential expression pattern similar for all five genes was not observed for individual samples. This heterogeneous differential expression pattern in DNMC when compared to MC across the 18 samples seems to be in line with the observations from the microarray expression analysis.

### 3.3. Proteome Analysis of Melanocytes from Dysplastic Naevus and Normal Skin

**Figure 3:**
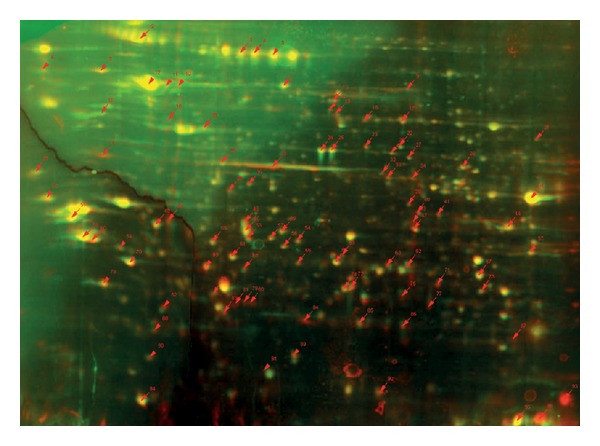
Representative 2D DIGE image of protein analysis. Red signal: overexpressed protein in DNMCs. Green signal: overexpressed protein in melanocytes from normal skin. Yellow signal: equal expression in both melanocyte types sample groups. Arrows indicate spots excised for MALDI-TOF/TOF analysis.

The 18 matched melanocyte sample sets, derived from either dysplastic naevus or normal skin, were additionally subjected to mass spectrometry analysis to assess whether protein expression differences existed between the melanocyte sample groups.  Figure 3 shows a representative example of proteins expressed in MC (visualized as green signal-Cy3) and the corresponding protein expression in DNMC (visualized as red signal-Cy5). Dye swap experiments to control for dye labelling efficiency performed on various samples indicated that no large differences in efficiency were present between both dyes (data not shown). Each of the 2D DIGE gels contained on average 2205 spots, 90% of these spots were reproducible in at least 15 gel sets. Protein spots (*n* = 99) that were differentially expressed between normal and atypical melanocytes in at least 15 gel sets determined by statistical analysis were excised and subsequent MALDI TOF/TOF analysis was performed. Database mining resulted in the annotation of 70 proteins (see Table 1 available as Supplementary Material online at doi:10.1155/2012/981308). Additional statistical analysis yielded 16 most differentially expressed proteins (*P* < 0.03, fold-change in expression difference ≥2 between DNMC and MC) (Table 3). 

Among these 16 proteins, there was decreased expression of vimentin and increased GTP-binding Rho-like protein CDC42 expression in the DNMC; these proteins are involved in regulation of cell shape and morphology. In addition, glutathione-S-transferase omega and tyrosine 3-monooxygenase, proteins involved in cellular detoxification processes, were found to be expressed at lower level in DNMC than in MC. Proteins that were involved in protein metabolism (prolyl 4-hydroxylase, eukaryotic translation initiation factor 3) we found to be higher expressed in DNMC.

 To assess whether overlap existed between the protein expression data and RNA expression data, we asked if for the 16 most differentially expressed proteins a similar expression pattern existed on the mRNA level. For each paired sample set of DNMC and MC, the corresponding gene expression fold-change was calculated. The fold-change in expression values was <1.5 for all probe sets and a consistent pattern in either increase or decrease in gene expression for a particular gene across all samples could not be observed (data not shown).

## 4. Discussion

In this study we compared the genome-wide gene expression and protein expression patterns of melanocytes derived from dysplastic naevus (DNMC) and melanocytes derived from adjacent normal skin (MC) from 18 patients. We aimed to find alterations in gene and protein expression that could inform us about biological processes underlying the earliest stage of melanoma development. Our results indicated that gene expression differences as measured using microarrays between melanocytes derived from normal skin and dysplastic naevus are rather small. Unbiased gene ontology analysis of the expression data of dysplastic naevus and normal melanocytes from a single patient enabled us to reveal differentially expressed GO classes; these included “organellar ribosome,” “mitochondrial ribosome,” “hydrogen ion transporter activity,” “prefoldin complex,” and “cAMP-dependent protein kinase regulator activity.” These GO classes reflect deregulated metabolic activity and growth of the DNMC as compared to MC, a factor that could contribute to the accumulation of cellular ROS levels.

We also aimed to determine differences in protein expression between the DNMC and melanocytes derived from normal skin. Comparison of the protein profiles between the two melanocyte sample groups resulted in 16 significantly differentially expressed proteins. Lower expression of cytoskeletal proteins such as vimentin and annexin in DNMC was observed, which is possibly associated with morphological alterations of DNMC observed in culture when compared to melanocytes derived from normal skin [7]. Proteins that were higher expressed in DNMC included eukaryotic translation initiation factor 3, GTP-binding Rho-like protein CDC42, prolyl 4-hydroxylase, platelet-activating factor acetylhydrolase, and DnaJ homolog subfamily B. These proteins are involved in protein synthesis, protein folding, and metabolism as well as lipid metabolism and cell shape organization [16, 17].

We did not observe an overlap of the 16 differentially expressed proteins with the microarray gene expression data, as a clear pattern in mRNA expression across all 18 sample sets was not found. This could be related to the overall small gene expression differences found in the microarray data. Dissimilarity between mRNA and protein abundance has been found in many combined genomic and proteomic studies. Nonetheless, among the differentially expressed proteins there were several involved in translation and growth, a functional class of proteins also identified in gene expression analysis.

For this study, melanocytes were isolated from patient biopsy samples and expanded in culture to obtain sufficient material for gene expression and proteome profiling. Cell culture, although short, will have impacted on mRNA and protein levels in these cells. However, the relative number of melanocytes present in the epidermis would be too small and variable across naevus and normal skin samples to allow for similar comparative analysis of whole tissue samples. Microdissection of the dysplastic naevus and normal skin biopsy samples prior to RNA and protein isolation would ensure isolated melanocyte populations, but it would not yield sufficient material for the type of microarray and proteomic analysis performed in the described study. The technology to isolate single melanocytic cells and perform whole transcriptome and proteome analyses is not yet available to us, if at all feasible currently. Evidently, a subset of preexisting biological differences in the transcriptomes and proteomes between the DNMC and MC may not be detected with the approach employed in this study, as the impact of culturing may overwhelm more subtle distinctions in expression. Any gene and protein expression differences however that may be induced by culturing, will be comparable between MC and DNMC since identical culture procedures have been followed for all DNMC and MC cultures in addition to harvesting cultures at the same passage numbers with the same harvesting methods. Moreover, we have compared DNMC and MC from the same patient from a single lesion and body site to maximize comparability. In spite of the impact of short-term culture on both types of cells, comparisons between these cells may be expected to yield meaningful results therefore.

For many gene and protein expression profiling studies established tumor cell lines and benign primary cells have often been comparatively analyzed. For this study early-passage cultures were used and comparisons were performed with benign cells of the same patient isolated from the vicinity of the lesional dysplastic cells. This approach, combined with the fact that expression patterns of dysplastic instead of malignant cells were compared to those of benign cells, probably also provides an explanation for the relatively limited range of expression differences we observed. 

For the melanocyte cultures, we made additional observations of representative MC and DNMC cultures in order to find out whether differences existed between the two melanocyte groups. Directly after plating of the epidermal single cell suspensions, the melanocytes from dysplastic naevus did not display differences in the rate of cell death or plate adherence as compared to the melanocytes from normal skin. In addition, DNMC and MC did not display significant differences in proliferation rate when seeded at equal densities at passage 4 in 6W plates and counted every day for 7 days. However, the DNMC cultures underwent senescence after prolonged periods in culture where normal MC cultures continued to proliferate. In addition modest but consistent differences in cellular morphology between DNMC and MC were observed, in particular pertaining to the shape of the dendrites. Together these findings are in agreement with previous electron microscopic studies revealing bloated mitochondria in DNMC and not in the MC as well as elevated ROS level measurements in DNMC when compared to MC [6, 7]. 

Until now, very little comparative gene expression and protein expression studies have focused on dysplastic naevi. Usually, melanocytes from either dysplastic naevi or normal skin are included in large expression studies aimed at later stages of melanoma development, involving melanoma samples [18–20]. In a comprehensive gene expression study that included biopsies from normal skin and dysplastic naevus, in addition to radial and vertical growth phase melanomas and melanoma metastases, it was observed that dysplastic naevi do not differ to a large extent from normal naevi based on gene expression profiles [21]. Even though the dysplastic naevi showed higher expression of proliferation-related genes compared to normal naevi and displayed similar molecular features to vertical growth phase melanomas, they displayed significant heterogeneity in gene expression profiles. Our results now indicate that differences between melanocytes from dysplastic naevus compared to melanocytes from normal skin are also modest.

Protein expression studies are to a similar extent often aimed at identifying changes occurring in late melanoma development stages; recently, it was found that 6.1% of dysplastic naevi nuclei versus 1.2% of normal, benign naevi nuclei stained positive for minichromosome maintenance (MCM) protein 2, a polypeptide belonging to the MCM family of proteins that are involved in DNA replication and are assumed to be prognostically useful as cell proliferation marker similar to Ki-67 [22]. In comparison, 49.1% nuclei of primary cutaneous melanomas stained positive. In addition, an earlier study found positive cyclin D1-nuclei staining in 0.34%, 5%, and 7.75% and positive cyclin D3-nuclei staining in 1.8%, 6.4%, and 17.8% in normal naevi, dysplastic naevi and melanoma biopsy sections, respectively [23]. These observations together indicate that expression differences of proteins that mark proliferating cells may also be limited in DNMC compared to MC whereas the most significant differences in protein expression remains when comparing dysplastic naevi with the primary melanoma stage.

A recent study however, found that the HLA class I heavy chain *β*2m and HLA class II*β* chain were expressed in 8.6% of the investigated normal, benign naevi and in 72% of atypical melanocytic lesions, where the level of expression correlated with degree of cytologic atypia and morphological disorder [24]. Further validation of a selected number of targets from our list of differentially expressed proteins could further confirm whether specific proteins also show significant difference in expression in DNMC compared to melanocytes from normal skin.

Since some dysplastic naevi bear more molecular similarity to their normal counterparts, whereas other dysplastic naevi more closely resemble more advanced primary melanoma in gene expression patterns [21], it may be not surprising that overall expression patterns of DNMC are heterogenous. Differences that do occur between DNMC and melanocytes from normal skin tissue may be specific and exclusive for only a subset of dysplastic naevus and potentially associated with the grade of atypia. The occurrence of such expression differences may also partially depend on history of formation of the original benign naevus; melanocytes from a dysplastic naevus that readily progresses to melanoma may have been more “primed” for transformation even before the initial naevus formation stage compared to melanocytes that may display characteristics of dysplasia, but are more restricted in their potential to progress to malignancy. It will be a challenge to design experimental approaches that can more accurately delineate gene expression alterations typical of dysplastic naevi, given the heterogeneity of their cellular composition and gene expression patterns. Novel single cell sequencing methodologies or sequencing of microdissected lesions may partially address some of these challenges.

Melanocytic cells are especially prone to oxidative DNA damage due to cellular metabolism processes such as melanin biosynthesis specific to these cells and exposure to UVA radiation. Deregulated expression of proteins involved in crucial oxidative stress management pathways in melanocytes of a dysplastic naevus can be detrimental and may contribute to their further progression towards malignancy. Recently exome analysis of melanoma has demonstrated that approximately 9% of driver mutations are G > T transversions reflecting the relevance of oxidative DNA damage as a mutagenic force in melanoma development [25]. Via a proteomic approach, we found several proteins differentially expressed in melanocytes from dysplastic naevus when compared to melanocytes from normal skin. Notably, proteins involved in the mitochondrial respiratory electron transport chain and overall oxidative phosphorylation process showed a decrease in expression in DNMC. A decrease in expression of proteins involved in oxidation reduction, cytoskeletal organization, and protein folding was also observed. Some of the proteins that showed significant higher level of expression in the DNMC were involved in protein metabolism, lipid oxidation, positive regulation of inflammatory responses, and protein refolding. The pattern of differential protein expression that our comparative analysis has yielded, may indicate that DNMC are indeed exposed to elevated levels of oxidative stress due to their own deregulated cellular metabolic processes, particularly mitochondrial oxidative phosphorylation, additionally suffering from faulty protein folding, and cytoskeletal organization. Which (epi)genetic alterations are causing oxidative stress to occur in melanocytes within a dysplastic naevus and whether these sets of alterations can serve to identify the dysplastic naevus cells are important issues that remain to be elucidated.

## 5. Conclusion

Genome-wide expression profiling of melanocytes derived from dysplastic naevus (DNMC) and melanocytes from normal skin (MC) resulted in significantly differentially expressed “organellar ribosome,” “mitochondrial ribosome,” “hydrogen ion transporter activity,” and “prefoldin complex” Gene Ontology classes, with overall gene expression differences between both melanocyte groups being relatively small. *MRPL42, PSMD10, HSPD1, MRPL47*, and *MRSP22* were among the top GO classes; validation of these genes showed a heterogeneous differential expression pattern across the DNMC and MC samples. Proteomic analysis revealed differentially expressed proteins in DNMC compared to MC with a role in cellular metabolism, detoxification, and cytoskeletal organization processes. Together these results indicate possible deregulated processes such as metabolism and protein synthesis in the melanocytes from dysplastic naevus; this is in line with the observed elevated oxidative stress levels that can potentially result in oxidative DNA damage in these cells. Further studies are needed to validate the identified differentially expressed proteins. Furthermore, investigations into which set of (epi)genetic alterations underlie the deregulated cellular processes that lead to increased oxidiatve stress, and whether these aberrations can specifically identify dysplastic naevus cells are warranted.

## Supplementary Material

Melanocytes derived from dysplastic naevus (DNMC) and normal adjacent skin (MC) of 18 different patients were subjected to mass spectrometry analysis in order to find proteins that were differentially expressed between both melanocyte sample groups. Mass fingerprint and MS/MS data were searched against the human protein database and resulted in the annotation of 70 proteins.

## Figures and Tables

**Table:** (a)

	GO category^1^	GO term^2^	GO description	Number of genes	LS permutation	KS permutation
					*P* value^3^	*P* value^4^
1	313	CC	Organellar ribosome	30	1.00*E* − 05	0.0120303
2	5761	CC	Mitochondrial ribosome	30	1.00*E* − 05	0.0120303
3	15078	MF	Hydrogen ion transporter activity	94	9.22*E* − 05	9.01*E* − 05
4	16272	CC	Prefoldin complex	13	0.0003082	0.001475
5	8603	MF	cAMP-dependent protein kinase regulator activity	9	0.0006046	0.0080679
6	4164	MF	Diphthine synthase activity	5	0.0006262	9.03*E* − 05
7	8629	BP	Induction of apoptosis by intracellular signals	17	0.0006292	0.0060299
8	79	BP	Regulation of cyclin-dependent protein kinase activity	30	0.0009682	0.0006918
9	8022	MF	Protein C-terminus binding	28	0.0010178	0.0332401
10	30274	MF	LIM domain binding	5	0.0027241	0.0278159
11	17182	BP	Peptidyl-diphthamide metabolism	8	0.0031029	0.001998
12	17183	BP	Peptidyl-diphthamide biosynthesis from peptidyl-histidine	8	0.0031029	0.001998
13	18202	BP	Peptidyl-histidine modification	8	0.0031029	0.001998
14	8428	MF	Ribonuclease inhibitor activity	5	0.0031816	0.0130247
15	45815	BP	Positive regulation of gene expression, epigenetic	7	0.0032192	0.0187543
16	43028	MF	Caspase regulator activity	5	0.0035432	0.3403788
17	31202	MF	RNA splicing factor activity, transesterification mechanism	23	0.0042451	0.0013586
18	8757	MF	S-adenosylmethionine-dependent methyltransferase activity	76	0.0044728	0.0618698
19	7021	BP	Tubulin folding	5	0.0046218	0.0028239
20	8013	MF	Beta-catenin binding	7	0.0048826	0.0031594
21	118	CC	Histone deacetylase complex	38	0.0080248	0.0020909
22	9897	CC	External side of plasma membrane	9	0.0085632	0.0021181
23	45736	BP	Negative regulation of cyclin-dependent protein kinase activity	5	0.0182021	0.0028634
24	3747	MF	Translation release factor activity	8	0.045947	0.0046691
25	8079	MF	Translation termination factor activity	8	0.045947	0.0046691
26	42393	MF	Histone binding	18	0.172388	0.0043185

^1^The 26 GO categories found to be significant at the nominal 0.005 level of the LS permutation test or KS permutation test (sorted by *P* values of the LS permutation test). For each GO category, the table lists the unique identifier, the number of genes in the project gene list that belong to the GO category, and the LS and KS *P* values.

The presented GO categories are ordered by the *P* value of the LS test (smallest first).

^2^Goterm: CC: cellular component, BP: biological process, and MF: molecular function.

^3^Fisher (LS) statistic is defined as the mean negative natural logarithm of the *P* values of the appropriate single gene univariate test.

^4^Kolmogorov-Smirnov (KS) statistic is used for testing if the *P* values are of a uniform distribution.

**Table:** (b)

	GO term^1^ Parametric	GO description	Probeset	Description^2^	Gene symbol	Parametric *P* values
1	CC	Organellar ribosome	219220_x_at	Mitochondrial ribosomal protein S22	MRPS22	0.0001097
2	CC	Organellar ribosome	223480_s_at	Mitochondrial ribosomal protein L47	MRPL47	0.000131
3	CC	Organellar ribosome	218558_s_at	Mitochondrial ribosomal protein L39	MRPL39	0.006304
4	CC	Organellar ribosome	224869_s_at	Mitochondrial ribosomal protein S25	MRPS25	0.0151834
5	CC	Organellar ribosome	222775_s_at	Mitochondrial ribosomal protein L35	MRPL35	0.0171902
6	CC	Mitochondrial ribosome	219220_x_at	Mitochondrial ribosomal protein S16	MRPS16	0.0005530
7	CC	Mitochondrial ribosome	223480_s_at	Mitochondrial ribosomal protein L44	MRPL44	0.000131
8	CC	Mitochondrial ribosome	219819_s_at	Mitochondrial ribosomal protein S28	MRPS28	0.0029398
9	CC	Mitochondrial ribosome	217919_s_at	Mitochondrial ribosomal protein L42	MRPL42	0.0303516
10	CC	Mitochondrial ribosome	222993_at	Mitochondrial ribosomal protein S37	MRPL37	0.003539
11	MF	Hydrogen ion transporter activity	213846_at	Cytochrome c oxidase subunit VIIc	COX7C	0.0020774
19	MF	Hydrogen ion transporter activity	218484_at	NADH dehydrogenase (ubiquinone) 1	NDUFA4L2	0.00585351
12	MF	Hydrogen ion transporter activity	238765_at	ATPase, H^+^ transporting, lysosomal 13 kDa, V1 subunit G1	ATP6V1G1	0.0059421
13	MF	Hydrogen ion transporter activity	209065_at	Ubiquinol-cytochrome c reductase-binding protein	UQCRB	0.0171665
14	MF	Hydrogen ion transporter activity	231487_at	Cytochrome c oxidase subunit 8C	COX8C	0.021483
15	MF	Hydrogen ion transporter activity	1552286_at	ATPase, H^+^ transporting, lysosomal 31 kDa, V1 subunit E2	ATP6V1E2	0.0279023
16	MF	Hydrogen ion transporter activity	202698_x_at	Cytochrome c oxidase subunit IV isoform 1	COX4I1	0.0380242
17	MF	Hydrogen ion transporter activity	228142_at	Ubiquinol-cytochrome c reductase complex (7.2 kD)	UCRC	0.0389511
18	MF	Hydrogen ion transporter activity	220834_at	Membrane-spanning 4-domains, subfamily A, member 12	MS4A12	0.0460068
20	CC	Prefoldin complex	207132_x_at	Prefoldin subunit 5	PFDN5	0.0086113
21	CC	Prefoldin complex	218336_at	Prefoldin subunit 2	PFDN2	0.0225808
22	CC	Prefoldin complex	222019_at	Prefoldin subunit 6	PFDN6	0.0326926
23	CC	Prefoldin complex	201472_at	Von Hippel-Lindau binding protein 1	VBP1	0.0345687
25	CC	Prefoldin complex	205963_s_at	DnaJ homolog, subfamily A, member 3	DNAJA3	0.04568034
26	MF	Oxidoreductase activity	207843_x_at	Cytochrome b5 type A	CYB5A	0.01245134
27	MF	Oxidoreductase activity	1560609_at	Crystallin zeta (quinone reductase)-like 1	CRYZL1	0.0496658

^1^The different GOterms: CC: cellular component; MF: molecular function; BP: biological process.

^2^Genes found to be differentially expressed with a parametric *P* value of less than 0.05 in the top 5 of GO categories.

They are ordered by the parametric *P* value associated with the GO category for each class.

**Table 2:** The 14 selected genes for validation of gene expression and qPCR primer sequences.

	Gene	GO description	Forward primer (5′–3′)	Reverse primer (5′–3′)
1	MRPS22	Organellar ribosome	GAACTGAAGCCACCAACCTATAAG	GCTGCCTCAACTGCCTGTC
2	MRPL42	Organellar ribosome	TCCAGTCCAAAATGGAGCTT	CCACAGAAGGGTGGTAGCAT
3	MRPL47	Organellar ribosome	ACCTGGTGCTTGGAGAAGAGAC	CACATAAGGCAAGGCAAAGAATCG
4	MRPL21	Organellar ribosome	GAGCCGAGATAGCTTCCTGA	CTCCTTCCCATTGGTTCTCA
5	MRPL7	Organellar ribosome	ACGCTTTGATTGCTCGATCT	TTGCCTCTTTGAAGCGTTTT
6	MRPL10	Organellar ribosome	GCAGAACAAGGAGCATGTGA	TTTCAGCCACCATGTCTTCA
7	MRPL17	Organellar ribosome	CACTCGTGAAACTGCTCAGG	CGTCGGAATGGTACACACTG
8	NDUFS1	Hydrogen ion transporter activity	TGCCCTAACCTCTAAGCCCTATG	ACTTCCAACCGCATCCATTACATC
9	NOX1	Hydrogen ion transporter activity	GCCTCCATTCTCTCCAGCCTATC	CACATACTCCACTGTCGTGTTTCG
10	ACADM	Mitochondrial	AGCCTTTACTGGATTCATTGTGG	ATTCCTCTAGTATCTGAACATCGC
11	HSPD1	Prefoldin complex	TACTGGCTCCTCATCTCACTCG	TGCTCAATAATCACTGTTCTTCCC
12	PSMF1	Prefoldin complex	AACACCTGGGTGACTTCCAC	CCCACTGCTCATGGATAGGT
13	PSMB9	Prefoldin complex	CGGGCGGGAGCACCAACC	GCAGACACTCGGGAATCAGAACC
14	PSMD10	Prefoldin complex	AGGTGCTCAAGTGAATGCTGTC	TGTAGCCTCATAATGGTCCTTAGC

**Table 3:** Top 16 differentially expressed proteins between melanocytes from dysplastic naevus and from normal skin.

Swiss-prot id	Protein name	Mol. mass calculated (Da)^1^	*p*_*I*_ obs.^2^	*p*_*I*_ calc.^3^	% Pept. Coverage^4^	Ratio (DNMC/MC)^5^	*P* value	General function
Q9Y2R9	Ribosomal protein S7	22113	10.09	10	43	0.1310	0.010946	Translational elongation
								
P32969	Ribosomal protein L9	21964	9.96	9.96	29	0.22334	0.016167	Translational elongation
								
P78417	Glutathione S-transferase omega 1-1	27833	6.23	6.24	21	0.23627	0.013815	Exhibits glutathione-dependent thiol transferase and dehydroascorbate reductase activities
								
P62753	Ribosomal protein S6	28842	10.9	10.85	23	0.31890	0.012139	Controlling cell growth and proliferation through the selective translation of particular classes of mRNA
								
O75947	ATP synthase, H^+^ transporting, mitochondrial F0 complex, subunit d	18537	5.21	5.21	41	0.32135	0.027965	Mitochondrial membrane ATP synthase that produces ATP from ADP
								
P04792	Heat shock protein 27	22427	7.83	5.98	57	0.36622	0.023047	Involved in stress resistance and actin organization
								
P02794	Ferritin heavy chain	21252	5.3	5.08	46	0.40067	0.006873	Stores iron in a soluble, nontoxic, readily available form. Important for iron homeostasis
								
Q59EQ2	Tyrosine 3-monooxy-genase/tryptophan 5-monooxygenase activation protein	28179	4.76	4.73	26	0.41836	0.012436	Oxidoreductase
								
P08670	Vimentin	53710	5.06	5.06	43	0.4478	0.023374	Vimentins are class-III intermediate filaments
								
P07355	Annexin A2	36631	8.32	7.56	53	0.47142	0.00642	May be involved in heat-stress response
								
P47985	Cytochrome b-c1 complex subunit Rieske	29934	8.55	6.3	5	0.47725	0.008578	Component of the ubiquinol-cytochrome c reductase complex
								
Q5U0F4	Eukaryotic translation initiation factor 3	36878	5.38	5.38	32	3.2155	0.018194	Protein synthesis
								
O94844	GTP-binding Rho-like protein cdc42	20123	5.25	4.83	55	2.88699	0.017896	The protein may play a role in small GTPase-mediated signal transduction and the organization of the actin filament system
								
P07237	Prolyl 4-hydroxylase	57480	4.76	4.69	46	2.79651	0.009987	This multifunctional protein catalyzes the formation, breakage, and rearrangement of disulfide bonds
								
Q6IBR6	Platelet-activating factor acetylhydrolase	25569	5.57	5.57		2.46963	0.008975	Lipid metabolic process
								
Q9UBS4	DnaJ (Hsp40) homolog, subfamily B	40774	5.81	5.81	30	2.33737	0.011076	Binds directly to both unfolded proteins that are substrates for ERAD and nascent unfolded peptide chains

^1^Calculated molecular mass of the protein in Dalton (Da).

^2^Isoelectric point (*p*_*I*_) observed in this study.

^3^ *p*_*I*_ calculated according to the Mascot database.

^4^Percentage peptide coverage.

^5^Protein ratio melanocytes from dysplastic naevus (DNMC)/melanocytes from normal skin (MC).
