# Supplementary material for: Genome-Wide Analysis of Gene and Protein Expression of Dysplastic Naevus Cells
Source: J Skin Cancer. 2012 Nov 28;2012:981308. doi: 10.1155/2012/981308 (PMC3515917; doi:10.1155/2012/981308)
Supplement: Supplementary file 1 — Melanocytes derived from dysplastic naevus (DNMC) and normal adjacent skin (MC) of 18 different patients were subjected to mass spectrometry analysis in order to find proteins that were differentially expressed between both melanocyte sample groups. Mass fingerprint and MS/MS data were searched against the human protein database and resulted in the annotation of 70 proteins. [file 981308.f1.doc]

**Supplementary Table I: Differentially expressed proteins in DNMC and MC**

1) Calculated molecular mass of the protein (Da)

2) Isoelectric point (pI) observed in this study

3) pI calculated according to the Mascot database

4) Percentage peptide coverage

5) General function of the protein

| **Swiss-prot id** | **Protein name** | **Mol. Mass calculated (Da)** ^1^ | **p*I* obs.**^2^ | **p*I* calc.**^3^ | **% Pept. Coverage**^4^ | **General function**^5^ |
| --- | --- | --- | --- | --- | --- | --- |
| **Metabolism** | |  |  |  |  |  |
| [Q9NR45](http://www.uniprot.org/uniprot/Q9NR45) | N-acetylneuraminic acid phosphate synthase | 40738 | 6,49 | 6,29 | 25 | Produces N-acetylneuraminic acid and 2-keto-3-deoxy-D-glycero-D-galacto-nononic acid. |
| [P37837](http://www.uniprot.org/uniprot/P37837) | TALDO1 protein | 37556 | 6,35 | 6,36 | 38 | Transaldolase is important for the balance of metabolites in the pentose-phosphate pathway. |
| Q15181 | Inorganic pyrophosphatase | 33095 | 5,54 | 5,54 | 46 | Phosphate metabolic process |
| A8K4W3 | Clathrin, light polypeptide A isoform a | 23704 | 4,45 | 4,45 | 30 | Intracellular protein transport |
| P78417 | Glutathione S-transferase omega 1-1 | 27833 | 6,23 | 6,24 | 21 | Exhibits glutathione-dependent thiol transferase and dehydroascorbate reductase activities |
| P07339 | Cathepsin D At Ph 7.5, Chain H | 26457 | 5,31 | 5,56 | 32 | Acid protease active in intracellular protein breakdown. |
| Q6IBR6 | Platelet-activating factor acetylhydrolase, isoform Ib, beta subunit | 25569 | 5,57 | 5,57 |  | Lipid metabolic process |
| P04792 | Heat shock protein 27 | 22427 | 7,83 | 5,98 | 57 | Involved in stress resistance and actin organization. |
| P63104 | 14-3-3 protein zeta | 25994 | 5,15 | 4,73 | 50 | Adapter protein implicated in the regulation of a large spectrum of both general and specialized signaling pathways. |
| P60174 | Triosephosphate isomerase | 26807 | 6,51 | 6,51 | 74 | Plays role in glycolytic and gluconeogenic metabolism |
| P52565 | Rho protein GDP-dissociation inhibitor 1 | 23250 | 5,02 | 5,03 | 55 | Regulates the GDP/GTP exchange reaction of the Rho proteins |
| P30048 | Peroxiredoxin 3 isoform b | 26107 | 7,04 | 7,68 | 18 | Involved in redox regulation of the cell. Protects radical-sensitive enzymes from oxidative damage by a radical-generating system. |
| Q15907 | RAB11B protein | 24591 | 5,46 | 5,65 | 44 | Possesses GTPase activity |
| P30085 | UMP-CMP kinase | 26180 | 8,41 | 8,75 | 31 | Catalyzes specific phosphoryl transfer from ATP to UMP and CMP. |
| P02794 | Ferritin heavy chain | 21252 | 5,30 | 5,08 | 46 | Stores iron in a soluble, non-toxic, readily available form. Important for iron homeostasis. |
| P22392 | Nucleoside-diphosphate kinase B | 17309 | 5,83 | 6,11 | 66 | Major role in the synthesis of nucleoside triphosphates other than ATP. |
| P00441 | Cu/Zn superoxide dismutase | 16020 | 8,76 | 6,74 | 12 | Destroys radicals which are normally produced within the cells and which are toxic to biological systems. |
| **Cytoskeletal** | |  |  |  |  |  |
| [Q9UQ80](http://www.uniprot.org/uniprot/Q9UQ80) | Proliferation-associated protein 2G4 | 41996 | 7,14 | 6,13 | 38 | Involved in ribosome assembly |
| P04632 | Calpain, small subunit 1 | 28453 | 5,05 | 5,05 | 43 | Regulatory subunit of the thiol-protease which catalyzes substrates involved in cytoskeletal remodeling and signal transduction. |
| O00562 | Phosphatidylinositol transfer protein | 31997 | 6,11 | 5,64 |  | Regulates RHOA activity, and plays a role in cytoskeleton remodeling. |
| [Q13748](http://www.uniprot.org/uniprot/Q13748) | Alpha-tubulin 2 isoform 8 | 46440 | 4,85 | 4,98 | 34 | Tubulin is the major constituent of microtubules. |
| [P08670](http://www.uniprot.org/uniprot/P08670) | Vimentin | 53604 | 5,09 | 5,06 | 30 | Vimentins are class-III intermediate filaments |
| [P08670](http://www.uniprot.org/uniprot/P08670) | Vimentin | 53710 | 5,06 | 5,06 | 43 | Vimentins are class-III intermediate filaments |
| [P60709](http://www.uniprot.org/uniprot/P60709) | Beta-actin | 41923 | 6,03 | 5,29 | 26 | Involved in various types of cell motility and are ubiquitously expressed in all eukaryotic cells. |
| P52907 | Capping protein alpha | 32955 | 5,58 | 5,45 | 16 | Binds to the fast growing ends of actin filaments thereby blocking the exchange of subunits at these ends. |
| P07355 | Annexin A2 | 36631 | 8,32 | 7,56 | 53 | May be involved in heat-stress response. |
| O15144 | Actin related protein 2/3 complex subunit 2 | 34426 | 6,84 | 6,84 | 22 | Functions as actin-binding component of the Arp2/3 complex which is involved in regulation of actin polymerization. |
| P47756 | F-actin capping protein beta subunit | 30952 | 5,69 | 5,36 | 49 | F-actin-capping proteins bind in a Ca(2+)-independent manner to the fast growing ends of actin filaments. |
| Q13637 | Low-Mr GTP-binding protein Rab32 | 24056 | 6,34 | 6,10 | 27 | Acts as an A-kinase anchoring protein by binding to the subunit of protein kinase A and anchoring it to the mitochondrion. |
| P13693 | IgE-dependent histamine-releasing factor | 19697 | 4,84 | 4,84 | 56 | Involved in calcium binding and microtubule stabilization. |
| P08729 | Keratin, type II cytoskeletal 7 | 51312 | 5,50 | 5,50 | 25 | Blocks interferon-dependent interphase and stimulates DNA synthesis in cells. |
| P23528 | Cofilin-1 | 18588 | 8,26 | 8,26 | 50 | Controls reversibly actin polymerization and depolymerization in a pH-sensitive manner. |
| **Protein processing** | |  |  |  |  |  |
| [Q99733](http://www.uniprot.org/uniprot/Q99733) | Nucleosome assembly protein 1-like 4 | 42823 | 4,60 | 4,60 |  | [Nucleosome assembly](http://www.ebi.ac.uk/ego/DisplayGoTerm?id=GO:0006334) |
| [P07237](http://www.uniprot.org/uniprot/P07237) | Prolyl 4-hydroxylase beta-subunit | 57480 | 4,76 | 4,69 | 46 | This multifunctional protein catalyzes the formation, breakage and rearrangement of disulfide bonds. |
| [Q9UBS4](http://www.uniprot.org/uniprot/Q9UBS4) | DnaJ (Hsp40) homolog | 40774 | 5,81 | 5,81 | 30 | Binds directly to both unfolded proteins that are substrates for ERAD and nascent unfolded peptide chains. |
| [Q5U0F4](http://www.uniprot.org/uniprot/Q5U0F4) | Eukaryotic translation initiation factor 3, subunit 2 beta, 36kDa | 36878 | 5,38 | 5,38 | 32 | [Protein biosynthesis](http://www.uniprot.org/keywords/648) |
| Q13765 | Nascent polypeptide-associated complex subunit alpha | 23370 | 4,52 | 4,52 | 37 | Prevents inappropriate targeting of non-secretory polypeptides to the endoplasmic reticulum (ER). |
| P07858 | Cathepsin B Chain C, Complexed With Dipeptidyl Nitrile Inhibitor | 29571 | 5,34 | 5,23 | 35 | Thiol protease which is believed to participate in intracellular degradation and turnover of proteins. |
| P28070 | Proteasome beta-subunit | 25950 | 5,70 | 5,47 | 41 | Cleavage of peptide bonds with very broad specificity. |
| P25786 | Proteasome alpha 1 subunit isoform 2 | 29822 | 6,15 | 6,15 | 30 | Cleavage of peptide bonds with very broad specificity. |
| Q99436 | Proteasome subunit beta type-7 | 27978 | 7,07 | 7,58 | 7 | Cleavage of peptide bonds with very broad specificity. |
| P62937 | Cyclophilin A | 18098 | 7,82 | 7,82 | 70 | PPIases accelerate the folding of proteins. |
| P25787 | Proteasome subunit alpha type 2 | 25865 | 7,12 | 6,91 | 34 | May have a potential regulatory effect on components of the proteasome complex through tyrosine phosphorylation. |
| Q06323 | Proteasome activator subunit 1 (PA28 alpha) | 28876 | 5,78 | 5,78 | 49 | Implicated in immunoproteasome assembly and required for efficient antigen processing. |
| P25787 | Proteasome subunit alpha type 2 | 25865 | 7,12 | 6,91 | 34 | May have a potential regulatory effect on components of the proteasome complex through tyrosine phosphorylation. |
| P11021 | Endoplasmic-reticulum-lumenal protein 28 | 29032 | 6,77 | 5,07 | 32 | Probably plays a role in facilitating the assembly of multimeric protein complexes inside the ER. |
| P62937 | Peptidyl-prolyl cis-trans isomerase A | 18209 | 7,44 | 7,82 | 59 | PPIases accelerate the folding of proteins. It catalyzes the cis-trans isomerization of proline imidic peptide bonds in oligopeptides. |
| **Vesicles** |  |  |  |  |  |  |
| P07900 | Heat shock protein HSP 90-alpha | 83445 | 5,00 | 4,94 | 40 | Molecular chaperone. Has ATPase activity |
| [P06748](http://www.uniprot.org/uniprot/P06748) | B23 nucleophosmin | 31090 | 4,71 | 4,64 | 27 | Functions in the assembly and/or transport of ribosome. |
| [PDIA3](http://www.uniprot.org/uniprot/B1MTI6) | Protein disulfide isomerase-associated 3 | 57146 | 5,98 | 5,77 | 32 | [Cell redox homeostasis](http://www.ebi.ac.uk/ego/DisplayGoTerm?id=GO:0045454) |
| [O14773](http://www.uniprot.org/uniprot/O14773) | Tripeptidyl-peptidase 1 | 39790 | 5,74 | 5,74 |  | Lysosomal serine protease with tripeptidyl-peptidase I activity. |
| [Q8N6T3](http://www.uniprot.org/uniprot/Q8N6T3) | ADP-ribosylation factor GTPase activating protein 1 | 44982 | 5,46 | 5,46 | 24 | Involved in membrane trafficking and /or vesicle transport |
| P40121 | Macrophage-capping protein | 38760 | 5,82 | 5,88 | 39 | Calcium-sensitive protein which reversibly blocks the barbed ends of actin filaments but does not sever preformed actin filaments. |
| P47985 | Cytochrome b-c1 complex subunit Rieske, mitochondrial | 29934 | 8,55 | 6,30 | 5 | Component of the ubiquinol-cytochrome c reductase complex |
| P32969 | Ribosomal protein L9 | 21964 | 9,96 | 9,96 | 29 | [Translational elongation](http://www.ebi.ac.uk/ego/DisplayGoTerm?id=GO:0006414) |
| P62753 | Ribosomal protein S6 | 28842 | 10,90 | 10,85 | 23 | Controlling cell growth and proliferation through the selective translation of particular classes of mRNA |
| O75947 | ATP synthase, H+ transporting, mitochondrial F0 complex | 18537 | 5,21 | 5,21 | 41 | Mitochondrial membrane ATP synthase that produces ATP from ADP |
| Q9Y2R9 | Ribosomal protein S7, cytosolic | 22113 | 10,09 | 10,00 | 43 | [Translational elongation](http://www.ebi.ac.uk/ego/DisplayGoTerm?id=GO:0006414) |
| Q13907 | Isopentenyl-diphosphate delta-isomerase 1 | 26645 | 5,93 | 5,93 | 35 | Catalyzes the 1,3-allylic rearrangement of the homoallylic substrate isopentenyl to dimethylallyl diphosphate |
| Q9BVK6 | Transmembrane emp24 domain-containing protein 9 | 25277 | 6,40 | 6,20 | 39 | Vesicle mediated transport |
| **Nuclear** |  |  |  |  |  |  |
| [Q14103](http://www.uniprot.org/uniprot/Q14103) | Heterogeneous nuclear ribonucleoprotein D isoform d | 30824 | 8,53 | 7,61 | 16 | Binds to double- and single-stranded DNA sequences in a specific manner and functions a transcription factor. |
| [Q56A79](http://www.uniprot.org/uniprot/Q56A79) | Purine-rich element binding protein A | 35003 | 6,29 | 6,29 | 22 | Probably playing a role in the control of both DNA replication and transcription |
| [O43432](http://www.uniprot.org/uniprot/O43432) | Eukaryotic translation initiation factor 4 gamma 3 | 29504 | 7,78 | 5,27 | 30 | Probable component of the protein complex eIF4F, which is involved in recruitment of mRNA to the ribosome. |
| P84103 | Splicing factor, arginine/serine-rich 3 | 19546 | 11,64 | 11,64 | 26 | May be involved in RNA processing in relation with cellular proliferation and/or maturation. |
| P31942 | Heterogeneous nuclear ribonucleoprotein H3 | 31505 | 6,76 | 6,37 | 17 | Involved in the splicing process and participates in early heat shock-induced splicing arrest. |
| **Unknown** |  |  |  |  |  |  |
| Q59GS1 | Ubiquitin carboxyl-terminal esterase L1 (Ubiquitin thiolesterase) variant | 25151 | 5,33 | n.a. | 72 | Evidence at transcript level. |
| O94844 | Gtp-binding rho-like protein cdc42, chain A | 20123 | 5,25 | 4,83 | 55 | The protein may play a role in small GTPase-mediated signal transduction and the organization of the actin filament system. |
| P63241 | Translation initiation factor eIF-5A | 17049 | 5,08 | 5,07 | 59 | The precise role of eIF-5A in protein biosynthesis is not known but it functions by promoting the formation of the first peptide bond. |
| Q59EQ2 | Tyrosine 3-monooxygenase/tryptophan 5-monooxygenase activation protein | 28179 | 4,76 | 4,73 | 26 | Evidence at transcript level. Oxidoreductase |
